# Rationale and design of the Exercise Intensity Trial (EXCITE): A randomized trial comparing the effects of moderate versus moderate to high-intensity aerobic training in women with operable breast cancer

**DOI:** 10.1186/1471-2407-10-531

**Published:** 2010-10-06

**Authors:** Lee W Jones, Pamela S Douglas, Neil D Eves, P Kelly Marcom, William E Kraus, James E Herndon, Brant A Inman, Jason D Allen, Jeffrey Peppercorn

**Affiliations:** 1 Duke University Medical Center, Durham, NC, USA; 2 University of British Columbia, Kelowna, British Columbia, Canada

## Abstract

**Background:**

The Exercise Intensity Trial (EXcITe) is a randomized trial to compare the efficacy of supervised moderate-intensity aerobic training to moderate to high-intensity aerobic training, relative to attention control, on aerobic capacity, physiologic mechanisms, patient-reported outcomes, and biomarkers in women with operable breast cancer following the completion of definitive adjuvant therapy.

**Methods/Design:**

Using a single-center, randomized design, 174 postmenopausal women (58 patients/study arm) with histologically confirmed, operable breast cancer presenting to Duke University Medical Center (DUMC) will be enrolled in this trial following completion of primary therapy (including surgery, radiation therapy, and chemotherapy). After baseline assessments, eligible participants will be randomized to one of two supervised aerobic training interventions (moderate-intensity or moderate/high-intensity aerobic training) or an attention-control group (progressive stretching). The aerobic training interventions will include 150 mins.wk^-1 ^of supervised treadmill walking per week at an intensity of 60%-70% (moderate-intensity) or 60% to 100% (moderate to high-intensity) of the individually determined peak oxygen consumption (VO_2peak_) between 20-45 minutes/session for 16 weeks. The progressive stretching program will be consistent with the exercise interventions in terms of program length (16 weeks), social interaction (participants will receive one-on-one instruction), and duration (20-45 mins/session). The primary study endpoint is VO_2peak_, as measured by an incremental cardiopulmonary exercise test. Secondary endpoints include physiologic determinants that govern VO_2peak_, patient-reported outcomes, and biomarkers associated with breast cancer recurrence/mortality. All endpoints will be assessed at baseline and after the intervention (16 weeks).

**Discussion:**

EXCITE is designed to investigate the intensity of aerobic training required to induce optimal improvements in VO_2peak _and other pertinent outcomes in women who have completed definitive adjuvant therapy for operable breast cancer. Overall, this trial will inform and refine exercise guidelines to optimize recovery in breast and other cancer survivors following the completion of primary cytotoxic therapy.

**Trial Registration:**

NCT01186367

## Background

Cardiorespiratory fitness is one of the most important indicators of health and longevity in humans [1,2]. The efficiency of mammals to transport O_2 _from the atmosphere to the muscle mitochondria determines an individual's cardiorespiratory fitness [3]. Maximal or peak oxygen consumption (VO_2peak_) provides the gold standard measurement of cardiorespiratory fitness and is strongly and inversely related to risk of death [4-8].

The importance of cardiorespiratory fitness for women following a breast cancer diagnosis has received limited attention[9]. However, over the past decade, there has been increased recognition and acceptance of the importance of therapy late effects (e.g., cardiovascular disease, type 2 diabetes, fatigue, deconditioning, etc.) as a major but underappreciated area in breast cancer management[10]. Emerging research evidence indicates that poor cardiorespiratory fitness may be of central importance for certain adverse late effects including impaired left ventricular ejection fraction, elevated cardiovascular disease (CVD) risk profile, poor quality of life, and fatigue following the completion of adjuvant therapy for operable breast cancer[11-13]. Further, recent landmark observational studies report that regular self-reported physical activity (e.g., brisk walking, ≥ 30 min^.^d^-1^, 5 d^.^wk^-1^), a major determinant of cardiorespiratory fitness, is associated with 30% to 50% reductions in breast cancer-specific mortality and all-cause mortality following the completion of adjuvant therapy[14,15].

Despite its importance, women with breast cancer have markedly reduced cardiorespiratory fitness. In a series of studies, we observed that cardiorespiratory fitness was approximately 30% below that of age-matched *sedentary *healthy women up to three years following the completion of adjuvant therapy[11,12,16]. The precise causes of poor cardiorespiratory fitness are not known but likely are a consequence of direct cytotoxic therapy-associated injury to the cardiovascular system (e.g., impairments in the organ components that govern VO_2peak_) together with lifestyle perturbations (e.g., deconditioning and weight gain) that we have termed the 'multiple hit' hypothesis[9].

A growing number of research groups have investigated the efficacy of supervised exercise training interventions (aerobic, resistance, or combination training) to counteract therapy-induced poor cardiorespiratory fitness both during and following the completion of adjuvant therapy. Overall, the current literature base indicates that supervised exercise training is a safe and feasible adjunctive therapy associated with significant improvements in objective measures of cardiorespiratory fitness as well as a broad range of patient-reported outcomes (PROs)[17]. Preliminary data also indicate that aerobic training may cause favorable improvements in circulating metabolic hormone concentrations in women with operable breast cancer following the completion of adjuvant therapy[18,19].

Although much progress has been made over the past 20 years, the format and intensity of exercise required to induce optimal improvements in cardiorespiratory fitness and other pertinent outcomes in women with breast cancer has not been investigated. The American College of Sports Medicine (ACSM) convened a panel of experts to review the available evidence supporting exercise prescription guidelines for cancer survivors[20]. The panel concluded that cancer survivors follow the 2008 Physical Activity Guidelines for Americans (≥ 150 mins.wk^-1 ^of moderate-intensity, or ≥ 75 mins.wk^-1 ^vigorous-intensity aerobic exercise or an equivalent combination of moderate- and vigorous intensity aerobic exercise) for cancer survivors, yet few studies have tested this empiric prescription in a formal randomized controlled trial. Furthermore, recent trials in other diseases besides cancer report that high-intensity exercise (≥ 70% of baseline VO_2peak_) is safe and feasible and associated with superior improvements in VO_2peak _and related endpoints relative to moderate-intensity training in patients with left ventricular dysfunction, heart failure, and metabolic syndrome[21-27]. To our knowledge, the efficacy of high-intensity exercise training has not been investigated in women with operable breast cancer.

Against this background, we designed the EXCITE trial, a randomized trial comparing the effects of supervised moderate-intensity to high-intensity aerobic training in women with operable breast cancer following the completion of primary adjuvant therapy. The primary aims are to: (1) compare the effects of high-intensity to moderate-intensity aerobic training, relative to attention-control, on VO_2peak_, (2) determine the effects on the physiological mechanisms that govern VO_2peak _(measurements of the heart-lung-skeletal muscle axis), and (3) to compare the effects on PROs. A secondary aim is to determine the effects on biomarkers associated with breast cancer recurrence that may underlie the exercise - breast cancer prognosis relationship (i.e., systemic metabolic hormones and cytokines/angiogenic factors). Herein we report details of the study design.

## Methods/Design

### Participants and Setting

In EXCITE, we will recruit and randomize 174 postmenopausal women (58 subjects/study arm) with histologically confirmed operable breast cancer (tumors positive or negative for estrogen receptors and progesterone receptors, and HER-2) following completion of primary therapy presenting at Duke University Medical Center (DUMC) for routine follow-up care will be eligible for this study. The DUMC institutional review board approved the study and written informed consent will be obtained from all participants prior to initiation of any study procedures. Additional inclusion and exclusion criteria are described in additional file 1.

### Procedures

**Figure 1:**
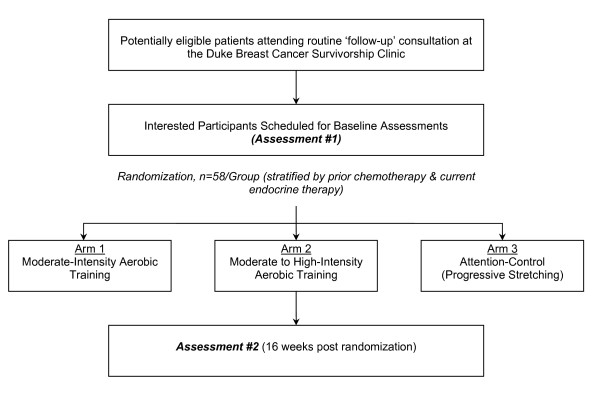
**Study Flow**.

The study flow is presented in Figure 1. Using a three-arm, randomized design, potential subjects will be identified and screened for eligibility by the study research coordinators via medical record review of patients scheduled to attend a 'follow-up' consultation at the Duke Breast Cancer Survivorship Clinic as per standard of care or following direct referral from their oncologist. Following primary attending oncologist approval, potential eligible subjects will be provided with a thorough review of the study and asked if they are willing to participate. After obtaining written consent, all participants will complete the following assessments in order of presentation: (1) fasting blood draw and oral glucose tolerance test, (2) peripheral vascular endothelial function, (3) spirometry and cardiopulmonary exercise test, and (4) echocardiogram at rest and during exercise and (5) PRO survey forms. On the following day, patients will undergo a tissue biopsy of the *vastus lateralis*. Participants will be asked to adhere to a water-only fast for 8 hours prior to testing on both days. All baseline assessments will be repeated at the end of the intervention (16 weeks). To maximize internal validity, study personnel, time of the day, equipment, and assessment order and timing will be the same at baseline and postintervention.

### Group Allocation (Randomization)

Following the successful completion of baseline assessments, participants will be randomly allocated, on an individual basis, to one of the two aerobic training interventions (high-intensity or moderate-intensity training) or an attention-control group (progressive stretching). Randomized participants will remain in the same group for the entire duration of the intervention. To ensure randomized groups are similar at baseline, patient randomization will be stratified based on prior treatment with chemotherapy and current treatment with endocrine therapy. A computer-generated list of random treatment assignments will be created by the trial statistician in sequentially numbered, opaque, sealed envelopes. A permuted block design with allocation weight of 1:1:1 will be used to generate the treatment assignments.

### Blinding and Masking

Study personnel conducting assessments at baseline and follow-up will be blinded to treatment assignment. Only the trial statistician and the data safety monitoring committee will have access to unblinded data, but none will have any direct contact with study participants or study personnel.

### Exercise Training Protocols (General Considerations)

The aerobic training interventions will include 150 mins.wk^-1 ^of supervised exercise sessions per week at an intensity of 60%-75% (moderate-intensity) or 60% to 100% (high-intensity) of the individually determined VO_2peak _between 20-45 min/session for 16 weeks. To achieve 150 mins.wk^-1 ^of supervised exercise per week, participants will be asked to exercise 4 times over a seven day period. Both aerobic training interventions are designed such that participants begin exercising at a low intensity (~50%-60% VO_2peak_) for shorter durations (~20-25 min/session) that is subsequently increased to a moderate (60%-70% VO_2peak_) or moderate-high intensity (60% to 100% VO_2peak_) for 150 mins.wk^-1 ^by week 4 of the program. All intervention sessions, including attention-control stretching, will be performed in a supervised setting with one-on-one supervision by a certified exercise physiologist for same duration per session to ensure that the only difference between sessions is the intensity of aerobic training. We believe that supervised sessions are critical to maximize adherence (>80% of planned sessions) to the intervention both in terms of attendance and adherence to the prescription. Optimizing adherence is critical to ensure a robust test of aerobic training and stretching (attention-control) efficacy.

#### Study Arm 1

Moderate-Intensity: Moderate-intensity aerobic training will be prescribed based on the ACS guidelines[20]. The moderate-intensity aerobic training intervention will work toward the target of four treadmill walking sessions/week at 60%-70% of VO_2peak _for 150 mins.wk^-1^. Treadmill walking was chosen because it is the preferred mode of exercise training for breast cancer patients[28].

In the introductory phase of the program (Weeks 1-4), the frequency, duration, and intensity of aerobic training will be progressively increased from an initial prescription of 3 session/wk for ~20 mins/session at ~60% of VO_2peak _to the ultimate goal of this prescription (150 mins.wk^-1 ^at 60%-70% VO_2peak_) at the end of week 4. The goal of these sessions will be to introduce aerobic training, including warm-up, heart rate and rate of perceived exertion monitoring, as well as cycling pace and proper form. Given that study participants will be sedentary, we believe that it is first important to introduce aerobic training and establish a cardiovascular base prior to introducing higher intensity aerobic training. Once the desired prescription has been obtained, in Weeks 5-16, patients will perform all sessions at the same intensity and duration for the remainder of the study consistent with the ACSM guidelines[20].

#### Study Arm 2

Moderate to High-Intensity Aerobic Training: The goal of this intervention is to improve VO_2peak_. As in Arm 1, all aerobic training sessions will be performed on a motorized treadmill. The moderate to high-intensity aerobic training intervention will target delivery of four treadmill walking sessions/week at 60%-100% of VO_2peak _for 150 mins.wk^-1^.

As in Arm 1, In the introductory phase of the program (Weeks 1-4), the frequency, duration, and intensity of aerobic training will be progressively increased from an initial prescription of 3 session/wk for ~20 mins/session at ~60% of VO_2peak _to the ultimate goal of this prescription (150 mins.wk^-1 ^at 60%-100% VO_2peak_) at the end of week 4. In Weeks 5 to 8 (intermediate phase), the goal will be to introduce higher intensity aerobic training. Specifically, exercise intensity will range between ~60%-70% VO_2peak _for two sessions per week; in the remaining two sessions, exercise intensity will be set at ventilatory threshold (~75% VO_2peak_). In weeks 9 to 16 (optimization phase), similar to the intermediate phase, participants will be asked to perform 4 aerobic sessions per week; 3 sessions will be performed at ~60%-80% VO_2peak_, and one interval workout. Interval workouts will consist of 2 min at the workload associated with peak VO_2 _(100% of VO_2peak_) followed by 2- 4 min of active recovery for 4-6 intervals. At week 8, participants will undergo a mid-point cardiopulmonary exercise test to re-assess VO_2peak_. On the basis of these results, the exercise prescription will be adjusted to ensure progressive improvements in VO_2peak _across the entire intervention.

#### Study Arm 3

Attention Control: Subjects assigned to the attention-control group will perform a supervised progressive stretching program that is matched to the exercise interventions in terms of program length 16 weeks), frequency [social interaction (participants will receive one-on-one instruction)], and duration (20-45 min per session). The progressive stretching program will be prescribed according the ASCM flexibility guidelines for older adults and aimed at increasing whole-body flexibility[29]. Stretching will be performed supine on stretching mats (no machines). Patients will be progressively trained to perform eight stretching exercises alternating between lower and upper body muscle groups.

##### Subject Retention/Adherence Considerations

To optimize subject retention and adherence in all three experimental conditions several strategies will be employed including: (1) individualized attention at the intervention sessions (in all experimental conditions), telephone calls following missed sessions, individuals meetings to outline goals and providing feedback on study progress, (2) participants will be allowed to make-up missed sessions within the 16-week study period, (3) participants will be allowed to schedule supervised interventions sessions at anytime from 7 AM to 7 PM, and (4) the PI and exercise physiologists will also meet on a weekly basis to review each participant's adherence with weekly intervention exercise prescription.

### Study Endpoints and Assessments

Table 1 outlines the study assessment schedule while a brief description of study endpoints and endpoint assessments including sub-studies is provided in additional file 2.

**Table 1:** Study Assessment Schedule

	Baseline					Post-Intervention (16 weeks)	
**Assessment**	**Screening**	**Day 0**	**Day 1**	**Day 2**	**Day 3-5**	**Day 112**	**Day 113**
Chart Review	x						
Patient Approached	x						
Informed Consent	x						
Outcome Assessments							
Patient-reported outcomes		x					
Blood draw		x				x	
Oral Glucose Test		x				x	
Endothelial Function		x				x	
VO_2peak_		x				x	
Echocardiogram		x				x	
Muscle biopsy			x				x
Repeat VO_2peak_				x			
Randomization				x			
Intervention initiation					x		

#### Primary Endpoint

To determine VO_2peak_, an incremental treadmill test (modified Balke protocol) with 12-lead ECG monitoring (Mac^® ^5000, GE Healthcare) will be performed by ACSM-certified exercise physiologists blinded to the patient's randomization group. Expired gases will be analyzed continuously by a metabolic measurement system (ParvoMedics TrueOne^® ^2400, Sandy, UT). After stable resting metabolic values have been achieved (including blood pressure and heart rate), subjects will begin walking at pace selected in the warm-up as a comfortable but brisk pace (~2.5-4.0 mph) at 0% grade for 2 minutes. Grade will be increased every 2 minutes until ventilatory threshold and then every minute until exhaustion or a symptom-limitation. During exercise oxyhemoglobin saturation will be monitored continuously using pulse oximetry (BCI, Hand-Held Pulse Oximeter, Waukesha, WN) while blood pressure will be measured every two minutes. Rating of perceived exertion will be evaluated at the end of each workload using the modified Borg Scale. Finally, significant learning effects are often observed in patient populations not familiar with CPET procedures. Thus, the CPET will be repeated prior to randomization to ensure the most accurate measurement of VO_2peak _and optimal aerobic training prescription.

#### Secondary Endpoints

*Physiological Determinants of VO_2peak _*will include pulmonary function, cardiovascular O_2 _delivery, peripheral artery endothelial function, and skeletal muscle function.

*Pulmonary Function *will be determined using standard spirometry. All measures will be performed in a sitting position according to the American Thoracic Society guidelines[30].

*Cardiovascular O_2 _Delivery *is comprised of four endpoints: *(1) Cardiac Output*. Left ventricular volumes and blood flow velocities will be performed with a commercially available ultrasound system (GE vivid-q BT10 System). Apical two- and four- chamber views will be assessed at rest and exercise to determine left ventricular end-diastolic volume and end-systolic volume by modified Simpson's rule[31,32]. Stroke volume will be calculated as end-diastolic volume minus end-systolic volume, which will be confirmed by measurement of left ventricular outflow tract area and the aortic flow velocity integral. Cardiac output will be calculated as stroke volume multiplied by heart rate. Blood pressure will be measured by auscultatory sphygmomanometry during exercise to determine mean arterial pressure. Systemic vascular resistance will be calculated as mean arterial pressure divided by cardiac output; *(2) Hemoglobin (Hb) Concentration *(O_2 _carrying capacity of blood) will be assessed via a venous blood draw according to standard guidelines, *(3) Arterial O_2 _Saturation *will be assessed at rest and continuously during exercise using pulse oximetry (Biox 3700, Ohmeda Medical, Boulder, CO), which provides the most accurate noninvasive assessment of blood arterial O_2 _saturation levels.

*Brachial Artery Endothelial Function *will be assessed using a commercially -available high-resolution ultrasound and a 7.5 MHz linear array transducer (HP Sonos 2000). Brachial artery assessments (a surrogate of systemic nitric oxide-bioavailability) will be obtained in longitudinal view, approximately 4 cm proximal to the olecranon process, in the anterior/medial plane, during the five minutes of forearm occlusion, and following cuff release (hyperemia) as previously described[33].

##### Skeletal Muscle Studies: Muscle biopsies and handling of tissue

Muscle will be obtained with the percutaneous needle biopsy technique (Bergstrom needles, DePuy Co., Inc., Warsaw, IN). We have consistently obtained tissue sample of 150-200 mg with a triple pass from a single incision, and we have demonstrated that this amount of tissue is sufficient for analyzing muscle fiber composition, capillarity, and metabolic enzymes[34].

##### Capillarity and histochemistry

The arrangement of capillaries in skeletal muscle typically is measured as capillaries^2^mm^-2 ^(capillary density). Capillary density is dependent on the size of the muscle fibers: larger fibers have lower capillary density. Thus, it is important to also express the extent of capillarization in terms of the capillary/fiber ratio, which is relatively independent of fiber area. Capillary counting will be performed as previously described[34]. For determination of fiber type, serial sections will be stained for myosin ATPase after preincubation at pH of 4.37, 4.6, and 10.4, to identify types I, IIa, and IIb muscle fibers[35].

##### Enzymology

The mitochondrial enzymes oxoglycerate dehydrogenase, citrate synthase, and 3-hydroxy-CoA-dehydrogenase will be determined. The activities of the glycolytic enzymes phosphofructokinase, aldolase, and lactate dehydrogenase will be measured as well as pyruvate dehydrogenase (total plus relative activity) as described previously[35].

*Patient Reported Outcomes *will include quality of life (QOL), fatigue, and depression. *QOL *will be assessed using the Functional Assessment of Cancer Therapy - Breast (FACT-B) scale developed for the assessment of patient symptoms and QOL in breast cancer patients[36]. *Fatigue *will be assessed using the 13-item FACT-fatigue scale for the assessment of fatigue in cancer patients[37]. Finally, *depression *will be assessed using the Center for Epidemiologic Studies Depression scale (CES-D)[38].

##### Biologic Mechanisms

*Whole-Body Metabolic Control *will consist of: *Insulin-Sensitivity *assessed using a standard oral glucose tolerance test analysis after an overnight fast. After sampling for baseline glucose and insulin, subject will be given a 296-mL glucose drink (Azer Scientific, Morgantown, PA) containing 75 g of glucose and samples drawn at 30, 60, 90, and 120 minutes after drink consumption. *Glycosylated Hemoglobin (HbA_1C_) *and *fasting insulin *will be measured using a CLIA approved clinical laboratory. *Systemic cytokines/angiogenic factors *will be evaluated and characterized in fasted blood prepared for multiplex assay analysis using Luminex 100/200 System (Luminex Corp, Austin, Tx) as previously described[39,40].

### Tracking and Monitoring of Adverse Events

Adverse events will be tracked and monitored using the following methods: (1) during intervention sessions, all patients will receive one-on-one supervision and all adverse events (e.g., knee pain, back pain) will be recorded on the patient case report form (CRF). In addition, heart rate, blood pressure, and O_2 _saturation will be recorded prior to, during, and following every intervention session, (2) at the beginning of each week, the exercise physiologist will spend the first 10 minutes of every session discussing any potential negative side-effects of the intervention assignment and any injuries that may have occurred; (3) during the program, all study participants will provide a blood sample every 4 weeks. A Complete Blood Count will conducted on each sample to monitor changes in white blood cell and white blood cell differential (neutrophils, lymphocytes, and monocytes) to provide insight into the effects of exercise on immune function, and (4) every six months a meeting of all investigators will be scheduled to review and discuss all reported non-serious and serious adverse events for early identification of negative issues and development of solutions. All serious adverse events will be immediately reported to DUMC IRB and immediately circulated to all study investigators for appropriate discussion, and early stopping rules in response to a differential higher frequency of adverse events in a particular study group will be enforced.

### Statistical Considerations

#### Sample Size Calculation

This randomized phase III trial will accrue 174 patients with operable breast cancer over an accrual period of ~48 months. The power of both an overall F-test comparison for the study's primary outcome variable, VO_2peak_, among experimental groups is considered as part of power calculations, as well as two primary pair wise comparisons (i.e., moderate-intensity versus moderate to high-intensity training; moderate to high-intensity training versus attention-control). The power of pairwise comparisons is computed with adjustment for multiple comparisons using a Bonferroni correction factor (α = 0.05/2 = 0.025). These calculations assume a lost-to-follow-up rate of 15% (8 in each group or 24 in total). Thus, it is anticipated that 150 will provide evaluable data at follow-up (16 weeks). The power estimates were calculated using the following observed/expected change in VO_2peak _from baseline to 16 weeks: moderate-intensity -training: +1.5 mL.kg.^-1^min^-1^; moderate to high-intensity training: +4.0 mL.kg.^-1^min^-1^; and attention-control: 0.0 mL.kg.^-1^min^-1^. Power estimates assume the SD for the change in VO_2peak _to be equal for all these groups (4.0 mL.kg.^-1^min^-1^) obtained from our prior work among post therapy breast cancer patients[11,12]. With 150 patients having evaluable post-intervention follow-up, there is >99% power to detect the noted difference among the three experimental groups with an F-test conducted with two degree of freedom. With 50 patients per group having evaluable post-intervention follow-up, there is 80% power to detect a 2.5 mL.kg.^-1^min^-1 ^difference in VO_2peak _between the aerobic training interventions; and >99% power to detect a 4.0 mL.kg.^-1^min^-1 ^difference in VO_2peak _between moderate to high-intensity aerobic training and attention-control using a two-sided alpha of 0.025 If analyses included all 174 patients (i.e. 58 per group), these pair-wise comparisons will have 86% and >99% power, respectively.

#### Analytic Plan

The principal analysis of the primary outcome will employ the intention-to-treat (ITT) approach. The ITT analysis will include all randomized participants in their randomly assigned group. The intervention group assignment will not be altered based on the participant's adherence to the randomly allocated study arm. The primary analysis will use a multiple regression model to test for differences among and between the study arms in VO_2peak _from baseline to the post intervention assessment (16 weeks). Specifically, two comparisons will be conducted: (1) moderate-intensity training versus attention control, and (2) moderate to high-intensity training versus attention control. The outcome variable in the basic regression model is change in VO_2peak_; whereas predictors include study group, baseline VO_2peak_, as well as the interaction. The regression model will allow controlling for the baseline value of the study endpoints and other confounding variables that may impact change in VO_2peak _(e.g., self-reported exercise history, age, prior treatment, exercise adherence). Finally, regression analyses will initially focus on participants that complete a follow-up assessment. Missing follow-up assessments will be imputed under different assumptions and used in additional analyses to assess the sensitivity of inferences to missing data. Multiple regression models will also be used to compare study arms relative to the change in physiological mediators (e.g. cardiac function, peripheral vascular endothelial function, and skeletal muscle oxidative capacity) of the aerobic training - VO_2peak _relationship. Following the guiding principles of Baron and Kenny, analyses will be conducted that investigate whether physiological variables, such as pulmonary function, cardiac output, and skeletal muscle function, mediate the effect of exercise training on VO_2peak_.

## Discussion

### Methodological Considerations

Several methodological issues were considered when designing EXCITE. Of these, the most paramount were issues surrounding the feasibility and tolerability of moderate to high-intensity aerobic training in women with a history of breast cancer. To our knowledge, all prior studies in breast cancer patients have investigated the effects of exercise training prescribed at a moderate-intensity (i.e., ~50% to ~70% baseline cardiorespiratory fitness) either during or following adjuvant therapy. There is a prevailing opinion in the oncology literature that high-intensity exercise training is not feasible or even contraindicated for individuals with a history of cancer;[41] this is likely based on the potential immunosuppressive effects of exercise training conducted ≥ 75% of VO_2peak_. However, evidence to support this notion is lacking. In contrast, recent trials in non-cancer clinical populations report that high-intensity exercise (≥ 70% of baseline VO_2peak_) is safe and well-tolerated approach associated with superior improvements in VO_2peak _and related endpoints, relative to moderate-intensity training[21-27]. Furthermore, two pilot studies by our group found that high-intensity aerobic training was feasible and safe among patients with non-small cell lung cancer[42,43]. Taken together, there exists sufficient evidence to investigate the efficacy of high-intensity aerobic training in women following completion of definitive adjuvant therapy for operable breast cancer. Given the novelty of this approach in the oncology setting, we will employ several methods to carefully monitor and assess adverse events in this trial as described earlier in the paper.

A second consideration is that adherence to supervised aerobic training 4 d^.^wk^-1 ^may be over burdensome for a proportion of women with breast cancer. The vast majority of prior exercise oncology trials have investigated the feasibility and efficacy of exercise training conducted 3 d^.^wk^-1^, which is clearly more feasible, but the goal of this study is to test the effects of ≥ 150 min of exercise per week, consistent with the ACSM recommendations[20]. Similarly, we acknowledge that while incorporating home-based sessions in the prescription may increase adherence, we believe that it critically important to monitor all exercise sessions to optimize safety and ensure that all sessions were performed at the correct intensity and ensure that the duration of each exercise session is equal between all groups.

## Conclusions

It is becoming increasingly apparent that cardiorespiratory fitness is a parameter of critical importance following a breast cancer diagnosis. However, as a result of the direct and indirect deleterious effects of anticancer therapy (the multiple-hit hypothesis), cardiorespiratory fitness is markedly reduced in breast cancer patients[3,9]. Although the long-term clinical implications are not known, such injury may increase susceptibility to competing causes of morbidity, poor quality of life, and even premature mortality

EXCITE was designed to investigate the intensity of aerobic training required to induce optimal improvements in VO_2peak _and other pertinent outcomes in women who have completed definitive adjuvant therapy for operable breast cancer. To the best of our knowledge, EXCITE is the first trial to compare the efficacy of different intensities of aerobic training and the first to examine changes in the physiologic mechanisms that govern cardiorespiratory fitness in breast cancer patients. The fundamental rationale for this study is that investigating the level and format of aerobic training that produces optimal improvements in VO_2peak _and other related clinical endpoints will inform evidence-based exercise guidelines for breast and other cancer populations. Insight into the changes in physiological determinants that underlie the aerobic training - VO_2peak _relationship will further refine exercise guidelines and inform novel combination approaches to maximize health and longevity in this population, as well as elucidating the mechanisms of benefit. Finally, the addition of correlative science studies that examine the effects of aerobic training on changes in biomarkers associated with recurrence/prognosis will inform mechanistically-driven clinical trials of exercise on surrogate markers of survival in breast cancer patients[41].

## Abbreviations

**EXCITE**: Exercise Intensity Trial; **DUMC**: Duke University Medical Center; **VO_2peak_**: Peak Oxygen Consumption; **CVD**: Cardiovascular Disease; **PROs**: Patient reported outcomes; **ACSM**: American College of Sports Medicine; **QOL**: Quality of Life; **FACT**: Functional Assessment of Cancer Therapy; **CRF**: Case Report Form; **IRB**: Institutional Review Board; **ITT**: Intention-to-Treat.

## Competing interests

The authors declare that they have no competing interests.

## Authors' contributions

All authors read and approved the final manuscript.

LWJ: conception and design and drafting of manuscript, PSD: conception and design, NDE: conception and design and drafting of manuscript, PKM: conception and design, WEK: conception and design.

JEH: conception and design, BAI: conception and design, JDA: drafting of manuscript, JP: conception and design and drafting of manuscript.

## Pre-publication history

The pre-publication history for this paper can be accessed here:

http://www.biomedcentral.com/1471-2407/10/531/prepub

## Supplementary Material

Additional file 1 **Additional Inclusion and Exclusion Criteria**. Table providing additional inclusion and exclusion criteria Click here for file

Additional file 2 **Study Measures**. Table describing study outcome measures. Click here for file

## References

[B1] Balady GJ Survival of the fittest--more evidence N Engl J Med 2002 346 11 852 854 10.1056/NEJM200203143461111 11893798

[B2] Kraus WE Douglas PS Where does fitness fit in? N Engl J Med 2005 353 5 517 519 10.1056/NEJMe058132 16079377

[B3] Jones LW Eves ND Haykowsky M Freedland SJ Mackey JR Exercise intolerance in cancer and the role of exercise therapy to reverse dysfunction Lancet Oncol 2009 10 6 598 605 10.1016/S1470-2045(09)70031-2 19482248

[B4] Gulati M Pandey DK Arnsdorf MF Lauderdale DS Thisted RA Wicklund RH Al-Hani AJ Black HR Exercise capacity and the risk of death in women: the St James Women Take Heart Project Circulation 2003 108 13 1554 1559 10.1161/01.CIR.0000091080.57509.E9 12975254

[B5] Myers J Prakash M Froelicher V Do D Partington S Atwood JE Exercise capacity and mortality among men referred for exercise testing N Engl J Med 2002 346 11 793 801 10.1056/NEJMoa011858 11893790

[B6] Ekelund LG Haskell WL Johnson JL Whaley FS Criqui MH Sheps DS Physical fitness as a predictor of cardiovascular mortality in asymptomatic North American men. The Lipid Research Clinics Mortality Follow-up Study N Engl J Med 1988 319 21 1379 1384 10.1056/NEJM198811243192104 3185648

[B7] Mora S Redberg RF Cui Y Whiteman MK Flaws JA Sharrett AR Blumenthal RS Ability of exercise testing to predict cardiovascular and all-cause death in asymptomatic women: a 20-year follow-up of the lipid research clinics prevalence study Jama 2003 290 12 1600 1607 10.1001/jama.290.12.1600 14506119

[B8] Sandvik L Erikssen J Thaulow E Erikssen G Mundal R Rodahl K Physical fitness as a predictor of mortality among healthy, middle-aged Norwegian men N Engl J Med 1993 328 8 533 537 10.1056/NEJM199302253280803 8426620

[B9] Jones LW Haykowsky MJ Swartz JJ Douglas PS Mackey JR Early breast cancer therapy and cardiovascular injury J Am Coll Cardiol 2007 50 15 1435 1441 10.1016/j.jacc.2007.06.037 17919562

[B10] Ganz PA Harnessing personalised medicine to prevent late effects Lancet Oncol 11 1 7 9 10.1016/S1470-2045(09)70344-4 19931488

[B11] Jones LW Haykowsky M Peddle CJ Joy AA Pituskin EN Tkachuk LM Courneya KS Slamon DJ Mackey JR Cardiovascular risk profile of patients with HER2/neu-positive breast cancer treated with anthracycline-taxane-containing adjuvant chemotherapy and/or trastuzumab Cancer Epidemiol Biomarkers Prev 2007 16 5 1026 1031 10.1158/1055-9965.EPI-06-0870 17507633

[B12] Jones LW Haykowsky M Pituskin EN Jendzjowsky NG Tomczak CR Haennel RG Mackey JR Cardiovascular reserve and risk profile of postmenopausal women after chemoendocrine therapy for hormone receptor--positive operable breast cancer Oncologist 2007 12 10 1156 1164 10.1634/theoncologist.12-10-1156 17962609

[B13] Herrero F Balmer J San Juan AF Foster C Fleck SJ Perez M Canete S Earnest CP Lucia A Is cardiorespiratory fitness related to quality of life in survivors of breast cancer? J Strength Cond Res 2006 20 3 535 540 10.1519/R-18215.1 16977706

[B14] Holmes MD Chen WY Feskanich D Kroenke CH Colditz GA Physical activity and survival after breast cancer diagnosis Jama 2005 293 20 2479 2486 10.1001/jama.293.20.2479 15914748

[B15] Irwin ML Smith AW McTiernan A Ballard-Barbash R Cronin K Gilliland FD Baumgartner RN Baumgartner KB Bernstein L Influence of pre- and postdiagnosis physical activity on mortality in breast cancer survivors: the health, eating, activity, and lifestyle study J Clin Oncol 2008 26 24 3958 3964 10.1200/JCO.2007.15.9822 18711185 PMC2654316

[B16] Haykowsky MJ Mackey JR Thompson RB Jones LW Paterson DI Adjuvant trastuzumab induces ventricular remodeling despite aerobic exercise training Clin Cancer Res 2009 15 15 4963 4967 10.1158/1078-0432.CCR-09-0628 19622583

[B17] McNeely ML Campbell KL Rowe BH Klassen TP Mackey JR Courneya KS Effects of exercise on breast cancer patients and survivors: a systematic review and meta-analysis Cmaj 2006 175 1 34 41 16818906 10.1503/cmaj.051073 PMC1482759

[B18] Irwin ML Varma K Alvarez-Reeves M Cadmus L Wiley A Chung GG Dipietro L Mayne ST Yu H Randomized Controlled Trial of Aerobic Exercise on Insulin and Insulin-like Growth Factors in Breast Cancer Survivors: The Yale Exercise and Survivorship Study Cancer Epidemiol Biomarkers Prev 2009 18 1 306 313 10.1158/1055-9965.EPI-08-0531 19124513 PMC2841479

[B19] Ligibel JA Campbell N Partridge A Chen WY Salinardi T Chen H Adloff K Keshaviah A Winer EP Impact of a mixed strength and endurance exercise intervention on insulin levels in breast cancer survivors J Clin Oncol 2008 26 6 907 912 10.1200/JCO.2007.12.7357 18281663

[B20] Schmitz KH Courneya KS Matthews C Demark-Wahnefried W Galvao DA Pinto BM Irwin ML Wolin KY Segal RJ Lucia A American college of sports medicine roundtable on exercise guidelines for cancer survivors Med Sci Sports Exerc 42 7 1409 1426 20559064 10.1249/MSS.0b013e3181e0c112

[B21] Rognmo O Hetland E Helgerud J Hoff J Slordahl SA High intensity aerobic interval exercise is superior to moderate intensity exercise for increasing aerobic capacity in patients with coronary artery disease Eur J Cardiovasc Prev Rehabil 2004 11 3 216 222 10.1097/01.hjr.0000131677.96762.0c 15179103

[B22] Hambrecht R Walther C Mobius-Winkler S Gielen S Linke A Conradi K Erbs S Kluge R Kendziorra K Sabri O Percutaneous coronary angioplasty compared with exercise training in patients with stable coronary artery disease: a randomized trial Circulation 2004 109 11 1371 1378 10.1161/01.CIR.0000121360.31954.1F 15007010

[B23] Giannuzzi P Temporelli PL Corra U Tavazzi L Antiremodeling effect of long-term exercise training in patients with stable chronic heart failure: results of the Exercise in Left Ventricular Dysfunction and Chronic Heart Failure (ELVD-CHF) Trial Circulation 2003 108 5 554 559 10.1161/01.CIR.0000081780.38477.FA 12860904

[B24] Wisloff U Stoylen A Loennechen JP Bruvold M Rognmo O Haram PM Tjonna AE Helgerud J Slordahl SA Lee SJ Superior cardiovascular effect of aerobic interval training versus moderate continuous training in heart failure patients: a randomized study Circulation 2007 115 24 3086 3094 10.1161/CIRCULATIONAHA.106.675041 17548726

[B25] Gordon A Tyni-Lenne R Jansson E Jensen-Urstad M Kaijser L Beneficial effects of exercise training in heart failure patients with low cardiac output response to exercise - a comparison of two training models J Intern Med 1999 246 2 175 182 10.1046/j.1365-2796.1999.00555.x 10447786

[B26] Duscha BD Slentz CA Johnson JL Houmard JA Bensimhon DR Knetzger KJ Kraus WE Effects of exercise training amount and intensity on peak oxygen consumption in middle-age men and women at risk for cardiovascular disease Chest 2005 128 4 2788 2793 10.1378/chest.128.4.2788 16236956

[B27] Tjonna AE Lee SJ Rognmo O Stolen TO Bye A Haram PM Loennechen JP Al-Share QY Skogvoll E Slordahl SA Aerobic interval training versus continuous moderate exercise as a treatment for the metabolic syndrome: a pilot study Circulation 2008 118 4 346 354 10.1161/CIRCULATIONAHA.108.772822 18606913 PMC2777731

[B28] Jones LW Courneya KS Exercise counseling and programming preferences of cancer survivors Cancer Pract 2002 10 4 208 215 10.1046/j.1523-5394.2002.104003.x 12100105

[B29] Nelson ME Rejeski WJ Blair SN Duncan PW Judge JO King AC Macera CA Castaneda-Sceppa C: Physical activity and public health in older adults: recommendation from the American College of Sports Medicine and the American Heart Association Circulation 2007 116 9 1094 1105 10.1161/CIRCULATIONAHA.107.185650 17671236

[B30] Kreider ME Grippi MA Impact of the new ATS/ERS pulmonary function test interpretation guidelines Respir Med 2007 101 11 2336 2342 10.1016/j.rmed.2007.06.019 17686622

[B31] Parisi AF Moynihan PF Feldman CL Folland ED Approaches to determination of left ventricular volume and ejection fraction by real-time two-dimensional echocardiography Clin Cardiol 1979 2 4 257 263 10.1002/clc.4960020404 262574

[B32] Schiller NB Acquatella H Ports TA Drew D Goerke J Ringertz H Silverman NH Brundage B Botvinick EH Boswell R Left ventricular volume from paired biplane two-dimensional echocardiography Circulation 1979 60 3 547 555 455617 10.1161/01.cir.60.3.547

[B33] Allen JD Cobb FR Gow AJ Regional and whole-body markers of nitric oxide production following hyperemic stimuli Free Radic Biol Med 2005 38 9 1164 1169 10.1016/j.freeradbiomed.2004.12.018 15808413

[B34] Duscha BD Kraus WE Keteyian SJ Sullivan MJ Green HJ Schachat FH Pippen AM Brawner CA Blank JM Annex BH Capillary density of skeletal muscle: a contributing mechanism for exercise intolerance in class II-III chronic heart failure independent of other peripheral alterations J Am Coll Cardiol 1999 33 7 1956 1963 10.1016/S0735-1097(99)00101-1 10362199

[B35] Kraus WE Torgan CE Duscha BD Norris J Brown SA Cobb FR Bales CW Annex BH Samsa GP Houmard JA Studies of a targeted risk reduction intervention through defined exercise (STRRIDE) Med Sci Sports Exerc 2001 33 10 1774 1784 10.1097/00005768-200110000-00025 11581566

[B36] Brady MJ Cella DF Mo F Bonomi AE Tulsky DS Lloyd SR Deasy S Cobleigh M Shiomoto G Reliability and validity of the Functional Assessment of Cancer Therapy-Breast quality-of-life instrument J Clin Oncol 1997 15 3 974 986 9060536 10.1200/JCO.1997.15.3.974

[B37] Yellen SB Cella DF Webster K Blendowski C Kaplan E Measuring fatigue and other anemia-related symptoms with the Functional Assessment of Cancer Therapy (FACT) measurement system J Pain Symptom Manage 1997 13 2 63 74 10.1016/S0885-3924(96)00274-6 9095563

[B38] Radloff LS The CES-D scale: A self report depression scale for research in the general population Applied Psychological Measurement 1977 1 385 401 10.1177/014662167700100306

[B39] Nikolinakos PG Altorki N Yankelevitz D Tran HT Yan S Rajagopalan D Bordogna W Ottesen LH Heymach JV Plasma cytokine and angiogenic factor profiling identifies markers associated with tumor shrinkage in early-stage non-small cell lung cancer patients treated with pazopanib Cancer Res 70 6 2171 2179 10.1158/0008-5472.CAN-09-2533 20215520 PMC4512950

[B40] Hanrahan EO Lin HY Kim ES Yan S Du DZ McKee KS Tran HT Lee JJ Ryan AJ Langmuir P Distinct patterns of cytokine and angiogenic factor modulation and markers of benefit for vandetanib and/or chemotherapy in patients with non-small-cell lung cancer J Clin Oncol 28 2 193 201 10.1200/JCO.2009.22.4279 19949019 PMC3040010

[B41] Jones LW Peppercorn J Exercise research: early promise warrants further investment Lancet Oncol 11 5 408 410 10.1016/S1470-2045(10)70094-2 20434709

[B42] Jones LW Eves ND Peterson BL Garst J Crawford J West MJ Mabe S Harpole D Kraus WE Douglas PS Safety and feasibility of aerobic training on cardiopulmonary function and quality of life in postsurgical nonsmall cell lung cancer patients: a pilot study Cancer 2008 113 12 3430 3439 10.1002/cncr.23967 18988290

[B43] Jones LW Peddle CJ Eves ND Haykowsky MJ Courneya KS Mackey JR Joy AA Kumar V Winton TW Reiman T Effects of presurgical exercise training on cardiorespiratory fitness among patients undergoing thoracic surgery for malignant lung lesions Cancer 2007 110 3 590 598 10.1002/cncr.22830 17582629

