# Supplementary material for: Rationale and design of the Exercise Intensity Trial (EXCITE): A randomized trial comparing the effects of moderate versus moderate to high-intensity aerobic training in women with operable breast cancer
Source: BMC Cancer. 2010 Oct 6;10:531. doi: 10.1186/1471-2407-10-531 (PMC2965727; doi:10.1186/1471-2407-10-531)
Supplement: Additional file 1 — Additional Inclusion and Exclusion Criteria. Table providing additional inclusion and exclusion criteria [file 1471-2407-10-531-S1.DOC]

Subject Eligibility Criteria

| Inclusion Criteria |  |
| --- | --- |
| At least 21 years old. |  |
| An interval of at least 1 year following the completion of primary therapy. | Primary therapy is defined as completion of surgery, radiotherapy, chemotherapy, or biologic (e.g., trastuzumab) therapy. We postulate that primary therapy causes acute unfavorable changes in cardiorespiratory fitness. Thus, to accurately determine the effects of different intensities of aerobic training on cardiorespiratory fitness in this setting, we felt it was important to initiate study procedures (i.e., recruit and randomize patients) ≥1 year following the completion of primary therapy to minimize the effects of natural (i.e., spontaneous) recovery on improvements in VO_2peak_ and other study outcomes, |
| Karnofsky performance status ≥70% at study entry |  |
| Estimated life expectancy of ≥6 months |  |
| Primary attending oncologist approval |  |
| Sedentary (i.e., patients not performing regular exercise on at least 3 days a week, for at least 30 minutes each session, at a moderate or vigorous intensity for the past month). This will ensure that only individuals not currently engaging in regular exercise (i.e., those who are the most likely to benefit) are recruited. |  |
| Willingness to be randomized, |  |
| Signed informed consent prior to initiation of study-related procedures |  |
| Current treatment with endocrine therapy (for women with endocrine positive tumors) is allowed. Given the potential effect of current therapy on study outcomes, endocrine therapy (yes / no) will be a stratification factor |  |
| Exclusion Criteria |  |
| Presence of a concurrent, actively treated other malignancy or history of other malignancy treated within the past 3 years (other than non-melanoma skin cancer), |  |
| Presence of metastatic disease |  |
| Subjects with any absolute contraindications to maximal exercise testing or aerobic training as recommended by the American Thoracic Society and exercise testing guidelines for cancer patients |  |
