# Supplementary material for: Rationale and design of the Exercise Intensity Trial (EXCITE): A randomized trial comparing the effects of moderate versus moderate to high-intensity aerobic training in women with operable breast cancer
Source: BMC Cancer. 2010 Oct 6;10:531. doi: 10.1186/1471-2407-10-531 (PMC2965727; doi:10.1186/1471-2407-10-531)
Supplement: Additional file 2 — Study Measures. Table describing study outcome measures. [file 1471-2407-10-531-S2.DOC]

| Study Measurements |
| --- |
| Physical measurements and tests |
| Height, mass, and body mass index |
| Resting and exercise heart rate |
| Resting and exercise blood pressure |
| Resting and exercise 12-lead ECG |
| Peak and submaximal oxygen consumption |
| Ventilatory threshold |
|  |
| Mechanistic physical measurements |
| Resting and exercise echocardiogram |
| Skeletal muscle function (fiber type distribution and oxidative capacity) |
| Hemoglobin concentration |
| Brachial artery reactivity – flow mediated dilitation |
| Resting and exercise oxygen saturation |
| Each intervention (exercise or attention control) session: heart rate, blood pressure, oxygen saturation, RPE |
|  |
| Patient-reported outcomes (questionnaires) |
| Medications |
| Quality of life |
| Fatigue |
| Depression |
| Adverse events |
|  |
| Systemic Biomarkers |
| Metabolic hormones |
| Cytokines and angiogenic factors |
| Complete blood counts |
|  |
